# Assessment of alteration in liver ^18^F–FDG uptake due to steatosis in lymphoma patients and its impact on the Deauville score

**DOI:** 10.1007/s00259-017-3914-y

**Published:** 2017-12-26

**Authors:** Thibault Salomon, Catherine Nganoa, Anne-Claire Gac, Christophe Fruchart, Gandhi Damaj, Nicolas Aide, Charline Lasnon

**Affiliations:** 1 Nuclear Medicine Department, Caen University Hospital, Caen, France; 2 Haematology Institute, Caen University Hospital, Caen, France; 3 Haematology Institute, François Baclesse Cancer Centre, Caen, France; 4 Normandie University, Caen, France; 5 INSERM 1086 ANTICIPE, Normandie University, Caen, France; 6 Nuclear Medicine Department, François Baclesse Cancer Centre, Caen, France

**Keywords:** Steatosis, Liver, FDG, PET, Lymphoma, Deauville score

## Abstract

**Aim:**

Our aim was (1) to evaluate the prevalence of steatosis in lymphoma patients and its evolution during treatment; (2) to evaluate the impact of hepatic steatosis on ^18^F–FDG liver uptake; and (3) to study how hepatic steatosis affects the Deauville score (DS) for discriminating between responders and non-responders.

**Methods:**

Over a 1-year period, 358 PET scans from 227 patients [122 diffuse large B cell lymphoma (DLBCL), 57 Hodgkin lymphoma (HL) and 48 Follicular lymphoma (FL)] referred for baseline (*n* = 143), interim (*n* = 79) and end-of-treatment (EoT, *n* = 136) PET scans were reviewed. Steatosis was diagnosed on the unenhanced CT part of PET/CT examinations using a cut-off value of 42 Hounsfield units (HU). EARL-compliant SUL_max_ were recorded on the liver and the tumour target lesion. DS were then computed.

**Results:**

Prevalence of steatosis at baseline, interim and EoT PET was 15/143 (10.5%), 6/79 (7.6%) and 16/136 (11.8%), respectively (*p* = 0.62).Ten out of 27 steatotic patients (37.0%) displayed a steatotic liver on all examinations. Six patients (22.2%) had a disappearance of hepatic steatosis during their time-course of treatment. Only one patient developed steatosis during his course of treatment. Liver SUL_max_ values were significantly lower in the steatosis versus non-steatotic groups of patients for interim (1.66 ± 0.36 versus 2.15 ± 0.27) and EoT (1.67 ± 0.29 versus 2.17 ± 0.30) PET. CT density was found to be an independent factor that correlated with liver SUL_max_, while BMI, blood glucose level and the type of chemotherapy regimen were not. Using a method based on this correlation to correct liver SUL_max_, all DS4 steatotic patients on interim (*n* = 1) and EoT (*n* = 2) PET moved to DS3.

**Conclusions:**

Steatosis is actually a theoretical but not practical issue in most patients but should be recognised and corrected in appropriate cases, namely, for those patients scored DS4 with a percentage difference between the target lesion and the liver background lower than 30%.

**Electronic supplementary material:**

The online version of this article (10.1007/s00259-017-3914-y) contains supplementary material, which is available to authorized users.

## Introduction

Steatosis or non-alcoholic fatty liver disease (NAFLD) is a hepatic complication of the metabolic syndrome and the major cause of hepatic abnormality throughout the world. It affects 10–24% of the general population in different countries [1]. NAFLD is histologically diagnosed by macrovesicular steatosis in >5% of hepatocytes. NAFLD comprises two main entities: simple steatosis or non-alcoholic fatty liver and non-alcoholic steato-hepatitis (NASH), which is characterised by lobular inflammation and ballooning degeneration.

Because of the prevalence of this disease, imaging diagnosis of hepatic steatosis has been evaluated as an alternative to invasive histological diagnosis. Several studies have demonstrated that liver attenuation on unenhanced computed tomography (CT) is an efficient tool to screen for moderate-to-severe hepatic steatosis, {corresponding to macrovesicular steatosis of 33% or greater [2]}, using a cut-off value of 42 Hounsfield units (HU) [3].

In the framework of treatment evaluation of diffuse large B cell lymphoma (DLBCL), follicular lymphoma (FL) and Hodgkin lymphoma (HL), liver uptake of ^18^F-FDG is taken as the reference tissue in assigning a Deauville score (DS), which underpins current criteria for interim PET and at the end of the treatment (EoT) PET response assessment. DS1–3 (less than or equal to liver background) versus D4–5 (greater than liver) are used to discriminate between responders and non-responders, respectively. This score has been shown to have a prognostic value early in the course of treatment and/or at the end of the treatment [4]. Importantly, visual assessment has to be confirmed by quantification, which is less user-dependent and circumvents optical misinterpretation due to the influence of background activity [5]. The use of liver background is based on previous studies that have determined that ^18^F-FDG uptake in the liver is relatively constant within the acquisition time specified by the EANM and SNMMI guidelines [6]. However, these statements were assessed in patients free of cancer and without any indication regarding the presence or not of hepatic steatosis. Therefore, extrapolation to current clinical practice could be challenged. To go further, this may lead to direct therapeutic implications when liver uptake is taken as the reference.

The aims of the present study were (1) to evaluate the prevalence of hepatic steatosis in lymphoma patients and its evolution across different time points (2) to evaluate the impact of hepatic steatosis on FDG liver uptake in the lymphoma population taking into consideration other known confounding parameters, especially body mass index (BMI) [7] and blood glucose level (BGL) [8]; and (3) to evaluate the potential impact of hepatic steatosis on therapeutic assessment according to the Deauville criteria.

## Materials and methods

### Patients’ recruitment

This study retrospectively included all patients over 18-years old diagnosed with HL, FL or DLBCL referred to our PET unit for baseline, interim after four cycles of chemotherapy and/or EoT examinations between November 2014 and December 2015. In accordance with European regulations, French observational studies without any additional therapy or monitoring procedure do not need the approval of an ethical committee. Nonetheless, we sought approval for our study from the national committee for data privacy, the National Commission on Informatics and Liberty (CNIL) with the registration n°2,080,317 v 0. All lines of treatment were considered. Consecutive patients referred by haematologists from our institution were identified by an automatic questionnaire on our picture archiving and communication system (PACS). The medical history of these patients was checked and only patients meeting inclusion criteria mentioned above and for whom international guidelines for PET tumour imaging had been fulfilled were included. For each patient, age, sex, international prognostic index (IPI or FLIPI), initial Ann Arbor staging, history of diabetes and liver dysfunction were recorded.

### PET-CT acquisition and reconstruction parameters

After a 15-min rest in a warm room, patients who had been fasting for 6 h were injected with ^18^F–FDG. The injected activity, capillary blood glucose level at injection time and the delay between injection and the start of the acquisition, height and body weight were recorded (acquisition parameters were extracted from the DICOM headers). Body Mass Index (BMI) was calculated as follows:

$$ BMI=\frac{body weight\ (kg)}{height^2\left({m}^2\right)} $$

All PET imaging studies were performed on a Biograph TrueV (Siemens Medical Solutions) with a 6-slice spiral CT component. Technical details regarding this system can be found elsewhere [9]. CT acquisition was performed first, with the following parameters: 60 mAs, 130 kV, pitch 1 and 6 × 2 mm collimation. Subsequently, the PET emission acquisition was performed in 3-D mode. Patients were scanned from the skull base to the mid-thighs, with time per bed acquisitions of 160 and 220 s for normal weight (BMI <25 kg/m^2^) and overweight patients (BMI ≥25 kg/m^2^), respectively.

Raw data were reconstructed with a PSF reconstruction algorithm (HD; TrueX, Siemens Medical Solution) with three iterations and 21 subsets and without filtering. Matrix size was 168 × 168, resulting in a 4.07 × 4.07 × 4.07 mm voxel size. Scatter and attenuation corrections were applied based on the CT scan.

### PET-CT analysis

All PET-CT examinations were reviewed on Syngo.via Software equipped with EQ.PET (Siemens Medical Solutions). A 6 mm Gaussian filter, determined as per the EARL accreditation program, was applied using the EQ.PET software [10].

For each PET-CT exam, liver maximum standardised uptake values (SUV_max_), lean body SUV_max_ (SUL_max_) and liver mean HU were measured using an automatic 3 cm-diameter volume of interest (VOI) set in the right liver lobe, avoiding liver lesions in the case of focal liver involvement.

Spleen mean HU was also recorded using a 2 cm-diameter VOI. Several cut-off values were used to define steatosis: mean liver HU ≤ 42, ratio between liver and spleen mean HU values (CT_L/S_) ≤ 0.8 and difference between liver and spleen mean HU values (CT_L-S_) ≤ −9 [3]. SUV_max_ and SUL_max_ in the mediastinum were measured in an automatically placed 1-cm diameter and 2-cm height cylinder in the descending thoracic aorta. In baseline examinations and in case of remaining lesions in interim and EoT examinations, the most intense target lesion was located by upscaling the base of the look up table on the 3D MIP view. SUV and SUL were computed as follows:

$$ {\displaystyle \begin{array}{c} SUV=\frac{measured activity\ \left(\frac{Bq}{cc}\right)\times body weight\ (g)}{injected dose\ (Bq)}\\ {} SUL=\frac{measured activity\ \left(\frac{Bq}{cc}\right)\times lean body mass\ (g)}{injected dose\ (Bq)}\end{array}} $$

The Deauville 5-point-scale (DS) was used to evaluate response for each interim and post-treatment PET/CT exam [11]:

DS1: No uptake
DS2: Uptake ≤ Mediastinum
DS3: Uptake > Mediastinum but ≤ Liver
DS4: Moderately increased uptake compared to the liver
DS5: Markedly increased uptake compared to the liver (defined as 2 times liver) and/or new lesions

## Statistical analysis

Quantitative data are presented as mean ± standard deviation (SD) or median (interquartile range) when appropriate. Characteristics of populations were compared by using Fischer’s exact tests or Chi-square tests for discrete variables and Mann-Whitney tests for continuous variables. Univariate and multivariate regressions were performed to determined parameters that affected liver SUV_max_ or SUL_max_. The difference between liver SUL_max_ in steatotic and non-steatotic patients was tested using the non-parametric Mann-Whitney test. Graphs and analyses were carried out using Prism version 5.0 (GraphPad Software, La Jolla, CA, USA) and MedCalc Statistical Software version 16.4.3 (MedCalc Software bvba, Ostend, Belgium). A *p*-value <0.05 was considered to denote statistical significance.

## Results

### Population characteristics and prevalence of steatosis in lymphoma patients

**Fig. 1:**
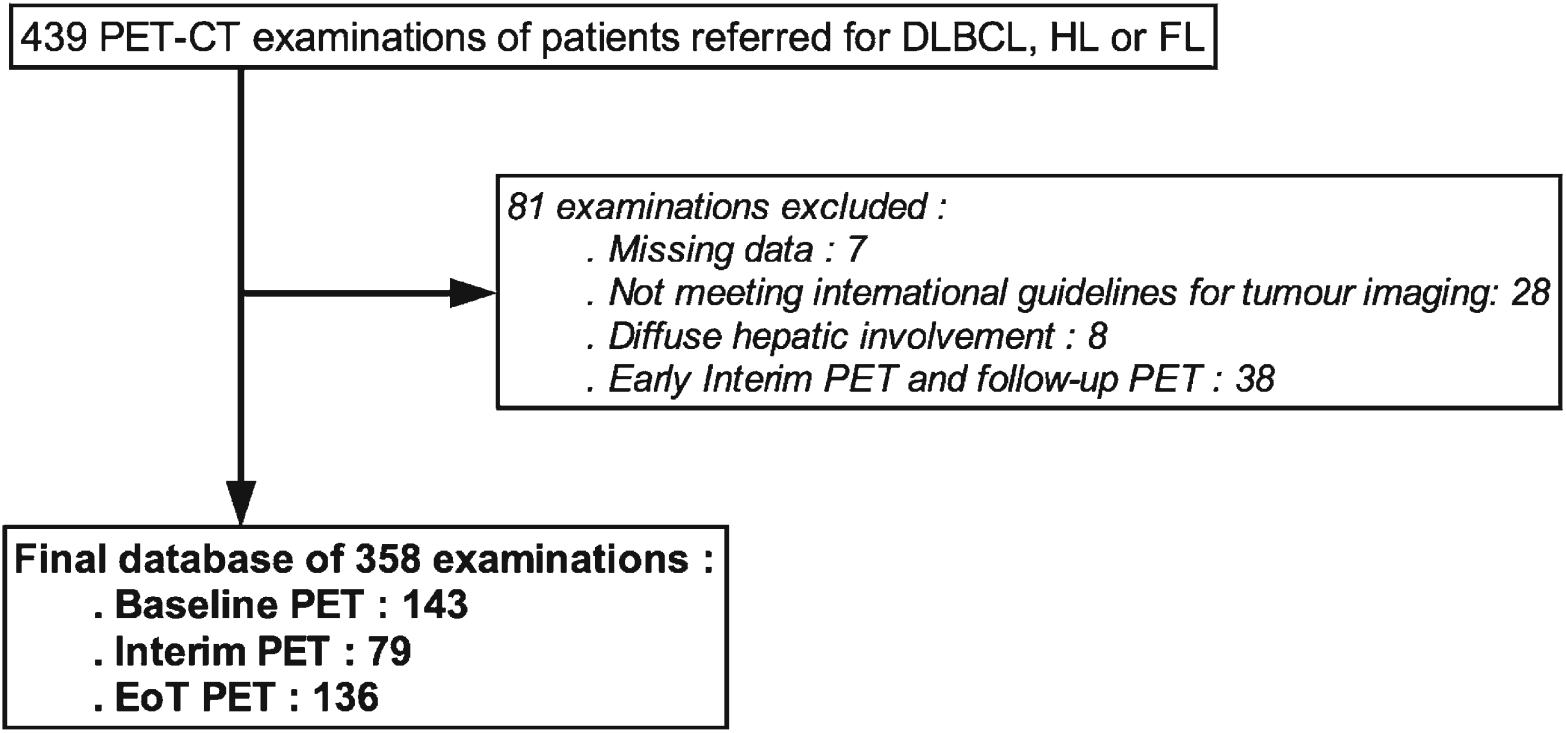
**Flow Chart of PET/CT examinations included in the study.** DLBCL: Diffuse large B cell lymphoma; HL: Hodgkin lymphoma; FL: follicular lymphoma; EoT PET: end of treatment PET

A total of 439 consecutive examinations were identified. After exclusion of PET examinations with missing data, not meeting international requirements for oncologic PET examinations, performed for other indications than baseline, interim or EoT therapeutic evaluation or demonstrating a diffuse hepatic involvement by disease, a final database of 358 PET-CT examinations in 227 patients was identified (Fig. 1). Over the time-period considered, 143 patients had a baseline PET-CT, 79 patients had an interim PET-CT and 136 patients had an EoT PET-CT. Prevalence of steatosis at baseline, interim and EoT PET was 15/143 (10.5%), 6/79 (7.6%) and 16/136 (11.8%), respectively (*p* = 0.62), when using HU_mean_ liver as diagnostic criterion. Using CT_L/S_ or CT_L-S_, frequencies were lower in all groups: eight (5.6%), four (5.1%) and 14 (10.2%) cases in baseline, interim and EoT PET groups, respectively, for CT_L/S_ and seven (4.9%), four (5.1%) and 11 (8.0%) cases in baseline, interim and EoT PET groups, respectively, for CT_L-S_. Noticeably, all patients identified by CT_L/S_ or CT_L-S_ were also identified by HU_mean_ liver criteria. As the use of HU_mean_ liver led to the higher number of cases, this criterion was used thereafter. With this criterion, 27 out of 227 patients (11.9%) demonstrated a steatotic liver on at least one of their PET-CT examinations.

**Fig. 2:**
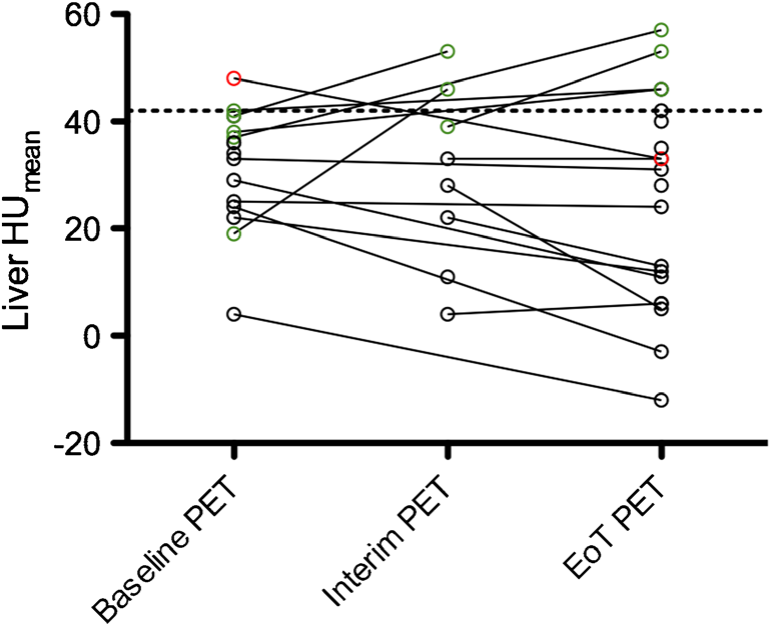
**Evolution of liver steatosis over the time-course of treatment.** All patients who had a steatotic liver on at least one of their examinations are displayed (*n* = 23). Noticeably, ten of them had only one PET-CT examination during the time-period considered. EoT: End Of Treatment, HU: Hounsfield Density

Patients’ characteristics can be found in detail in Table 1. The time course of the liver density (HU_mean_) of patients who demonstrated a steatotic liver on a least one of their PET-CT examinations is displayed in Fig. 2. Most of the steatotic patients (*n* = 10) displayed a steatotic liver on all of their examinations. Six patients had a disappearance of hepatic steatosis during their time-course of treatment. Noticeably, only one patient developed steatosis during his course of treatment. He was an overweight 68-year old man (BMI = 34.5 kg/m^2^) with no history of diabetes or liver dysfunction, diagnosed with a DLBCL (Ann Arbor stage IV, IPI 2), who underwent R-CHOP as a first line of treatment and was staged Deauville score (DS) 1 on the EoT PET-CT.

**Table 1:** Population characteristics

Characteristics	Steatotic patients (*n* = 27)	Non steatotic patients (*n* = 200)	*P* value
Age (yr), mean ± SD [min-max]	59.8 ± 17.2 [20–85]	56.1 ± 17.4 [17–88]	0.2622
Sex, n (%)			
Female	15 (55.6)	85 (42.5)	0.2201
Male	12 (44.4)	115 (57.5)	
BMI (kg/m^2^), mean ± SD	32.2 ± 7.6	24.4 ± 4.3	< 0.0001
Diabetes, n (%)			
Yes	2 (7.4)	5 (2.5)	0.1967
No	25 (92.6)	195 (97.5)	
Histologic type, n (%)			
DLBCL	16 (59.3)	106 (53.0)	0.4069
HL	4 (14.8)	53 (26.5)	
FL	7 (25.9)	41 (20.5)	
Ann Arbor Stage, n (%)			
I	6 (22.2)	27 (13.5)	0.4413
II	5 (18.5)	54 (27.0)	
III	3 (11.1)	35 (17.5)	
IV	13 (48.1)	84 (42.0)	
IPI, n (%)			
0–1	6 (37.4)	48 (45.3)	0.7689
2	4 (25.0)	27 (25.4)	
3–4-5	5 (31.3)	25 (23.6)	
*n.a*	*1 (6.3)*	*6 (5.7)*	
FLIPI, n (%)			
0–2	5 (71.4)	25 (61.0)	0.7079
3–5	2 (28.6)	14 (34.1)	
*n.a*	*0 (0.0)*	*2 (4.9)*	
Line of treatment, n (%)			
First-line	20 (74.1)	162 (81.0)	0.3968
Others	7 (25.9)	38 (19.0)	

*BMI* body mass index, *DLBCL* diffuse large B cells lymphoma, *HL* Hodgkin lymphoma, *FL* follicular lymphoma, *IPI* international prognosis index, *FLIPI* follicular lymphoma IPI, *n.a* not applicable

Steatotic patients had higher BMI (32.2 ± 7.6 kg/m^2^ versus 24.4 ± 4.3 kg/m^2^, *p* < 0.0001) than the non-steatotic group. Others clinical characteristics were not significantly different between these groups. Focusing on the 164 patients who underwent interim and/or EoT PET-CT (18 steatotic patients and 146 non-steatotic patients), there was a significant difference between administered treatments. Steatotic patients received (R-) CHOP, ABVD and other treatments in 33.3%, 5.6% and 61.1% of cases, respectively. Non-steatotic patients received (R-)CHOP, ABVD, BEACOPP, (R-)ACVBP or other treatments in 48.6%, 12.3%, 11.7%, 7.5% and 19.9% of cases, respectively (*p* = 0.0029).

### Relationship between steatosis and liver uptake on interim and EoT PET scans

Liver SUV_max_ values were highly correlated with BMI in both interim and EoT groups with an R^2^ value equal to 0.25 (*p* < 0.0001) and 0.27 (*p* < 0.0001), respectively. To remove the influence of BMI on liver uptake values that appeared to be a confounding factor regarding steatosis (see previous section), subsequent analyses were conducted using SUL_max_. Using SUL_max_ values instead of SUV_max_ values, one patient (1.3%) was staged Deauville score (DS) 3 instead of DS 2 in the interim group and one patient (0.7%) was staged DS 4 instead of DS 5 in the EoT group.

**Fig. 3:**
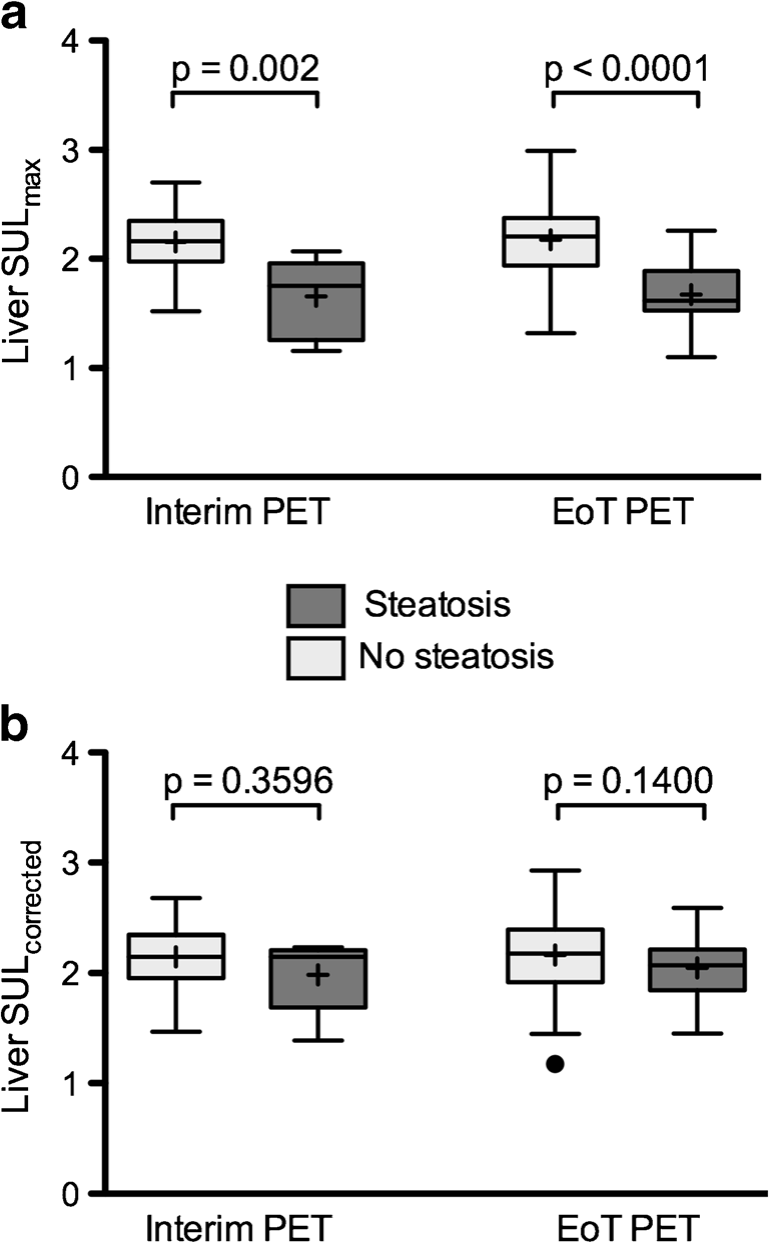
**Impact of steatosis on liver uptake.** (**a**) Liver SUL_max_ values of steatotic and non-steatotic patients before correction regarding liver density/steatosis. (**b**) Same data after correction. Data are shown as Tukey boxplots (lines displaying median, 25th and 75th percentiles; cross represents the mean value). EoT: End of Treatment

**Fig. 4:**
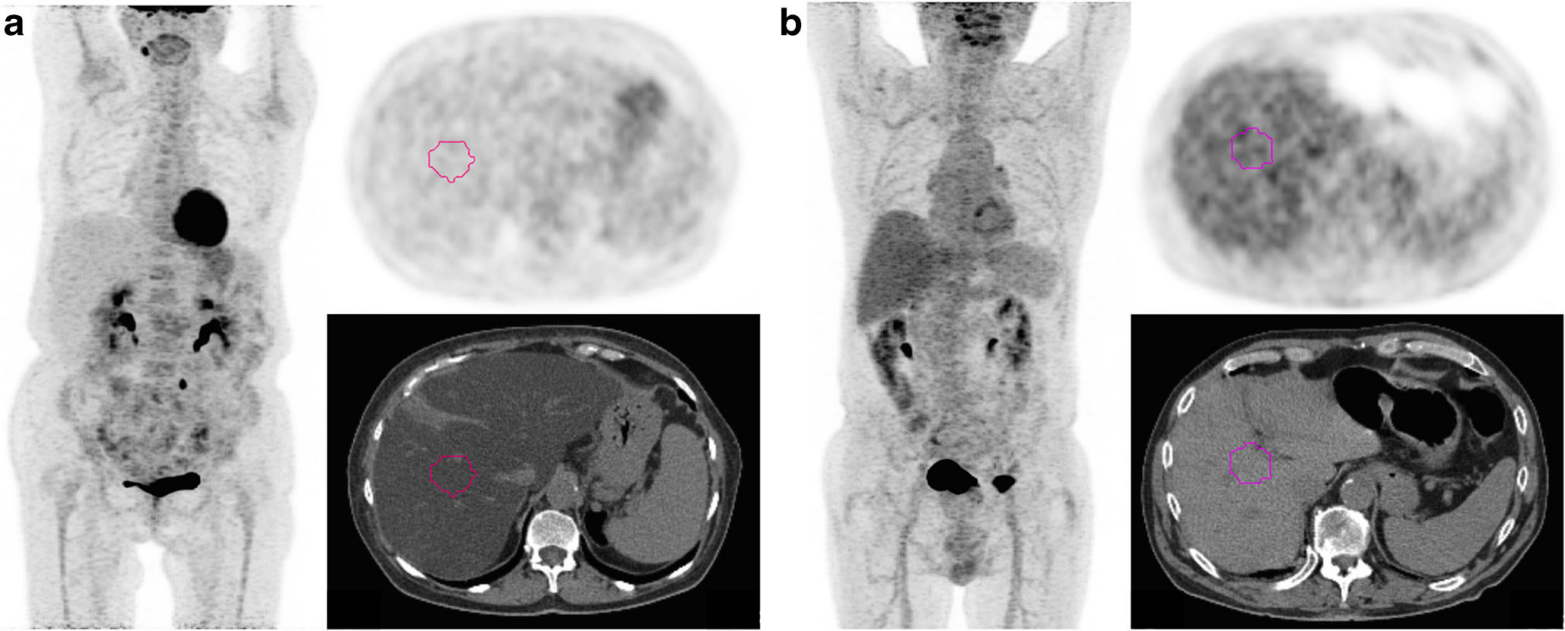
**Representative examples of steatotic (a) and non-steatotic (b) patients.** Maximum intensity projection (MIP) and trans-hepatic axial PET and CT images of 67 year-old female steatotic patient addressed for an interim PET of DLBCL scored DS5 (**a**) and a 84-year-old male non-steatotic patient addressed for EoT PET of a DLBCL scored DS1 (**b**). The automatic 3 cm-diameter VOIs in the right liver lobe are displayed in pink. Images are scaled on the same maximum value. Note that the intense FDG focus on the left groin of the patient illustrated on panel **b** is a benign uptake due to a plug

Mean liver SUL_max_ values were significantly lower in the steatotic versus non-steatotic groups of patients for both interim and EoT PET: 1.66 ± 0.36 versus 2.15 ± 0.27 and 1.67 ± 0.29 versus 2.17 ± 0.30, respectively (Fig. 3a). Liver SUL_max_ values were significantly correlated with liver HU_mean_ values in interim PET (*R* ^2^ = 0.1695, *p* = 0.0002) and in EoT PET (*R* ^2^ = 0.1671, *p* < 0.0001) (Table 3). Fig. 4 displays representative examples of steatotic and non-steatotic patients. The type of treatment did not appear to impact liver SUL_max_ in the interim and EoT PET-CT examinations. At the EoT time-point, BGL appeared to be significantly higher in examinations displaying a steatotic liver as compared to those with normal liver density (Table 2). However, it was not significantly correlated with liver SUL_max_ values for both interim and EoT PET-CT (Table 3). BMI was inversely correlated to liver SUL_max_ values only in the EoT group (*p* = 0.00154, *R* ^2^ = 0.0430) but, by multivariate analysis, liver HU_mean_ value was the only independent parameter associated with liver SUL_max_ values (R partial = 0.3607, *p* < 0.0001) (Table 3).

**Table 2:** PET-CT examinations characteristics

Characteristics	PET examinations with liver HU_mean_ ≤ 42	PET examinations with liver HU_mean_ > 42	*P* value
BGL (g/l), mean ± SD			
Interim PET	1.08 ± 0.24	0.98 ± 0.16	0.5976
EoT PET	1.10 ± 0.20	0.98 ± 0.14	0.0319
Injected dose (MBq/kg), mean ± SD			
Interim PET	3.95 ± 0.12	3.97 ± 0.19	0.7812
EoT PET	3.94 ± 0.16	4.00 ± 0.18	0.4059
Post-injection time (min), mean ± SD			
Interim PET	61.5 ± 3.5	59.9 ± 4.0	0.2129
EoT PET	60.6 ± 4.7	60.2 ± 4.0	0.9756

*BGL* blood glucose level, *HU* Hounsfield density, *EoT* end of treatment

**Table 3:** Relationship between liver SUL_max_ and liver HU_mean_ and identified confounding parameters (BGL, BMI, treatment type) by univariate and multivariate linear regression analyses

	Univariate analysis		Multivariate analysis	
SUL_max_ vs.	R^2^	*P* value	R partial	*P* value
Interim PET-CT examinations (*n* = 79)				
Liver HU_mean_	0.1695	0.0002	–	–
BGL	0.0005	0.8489	–	–
BMI	0.0193	0.2215	–	–
Treatment type	0.0063	0.4873	–	–
EoT PET-CT examinations (*n* = 136)				
Liver HU_mean_	0.1671	< 0.0001	0.3607	< 0.0001
BGL	0.0188	0.1117	–	–
BMI	0.0430	0.0154	0.0222	0.7986
Treatment type	0.0048	0.4248	–	–

*BMI* body mass index, *BGL* blood glucose level, *HU* Hounsfield density, *EoT* end of treatment

### Method to adjust liver SUL_max_ values in steatotic patients

**Fig. 5:**
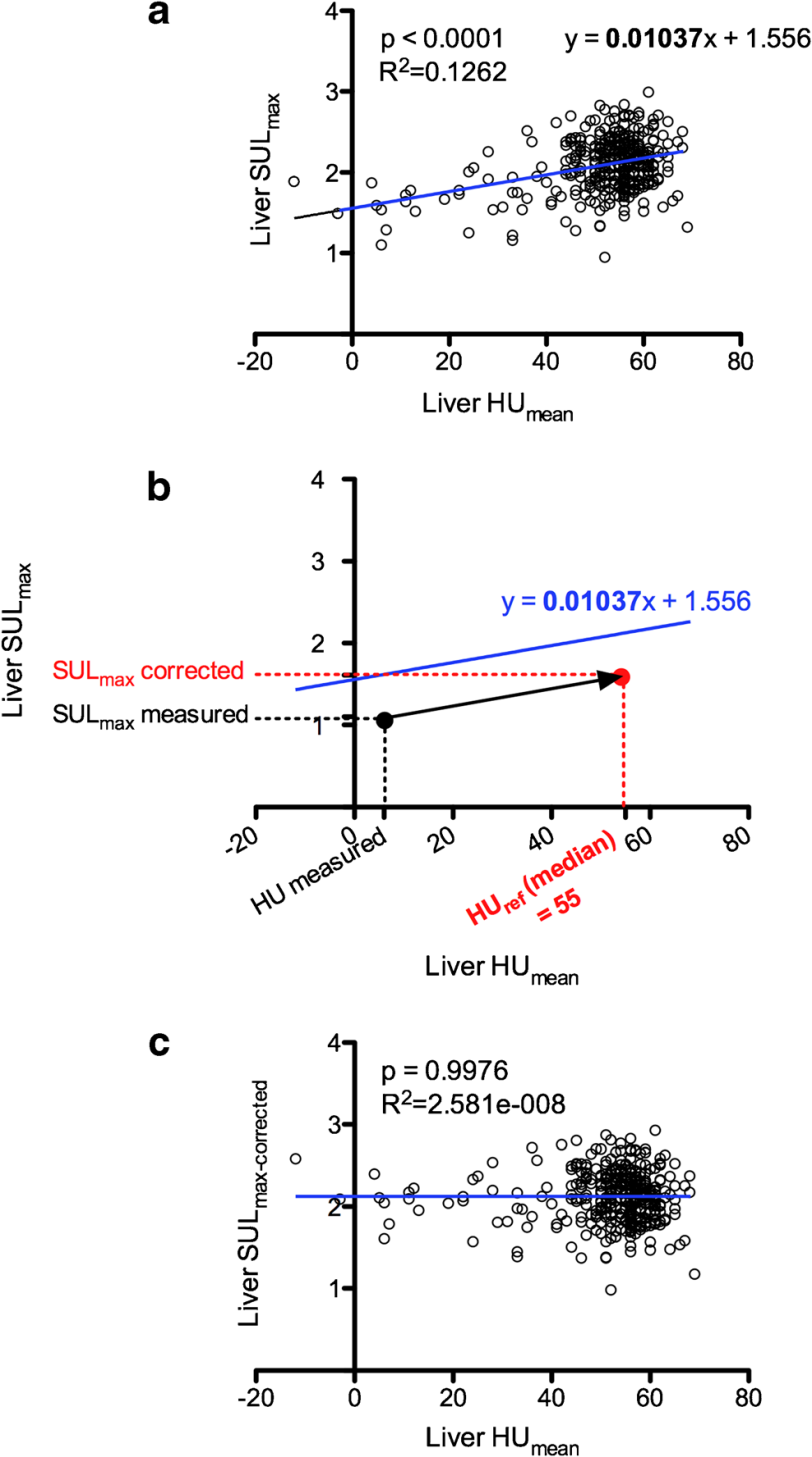
**Illustration of the method used for liver SUL**_**max**_ **correction regarding liver density/steatosis.** (**a**) Correlation between liver SUL_max_ values and liver HU_mean_ values of all PET-CT examinations before correction. (**b**) Illustration of the graphical method used for the correction of liver SUL_max_ values regarding liver density/steatosis. (**c**) Correlation between liver SUL_max_ values and liver HU_mean_ values of all PET-CT examinations after correction

Liver SUL_max_ were corrected using a graphical method based on the slope of the linear regression equation observed between liver HU_mean_ and liver SUL_max_ values of all PET-CT examinations (Fig. 5a). For all examinations considered, the median value of liver HU_mean_ was 55 HU. This value was then taken as the value of reference: HU_ref_.

The equation for the adjustment of SUL_max_ values is as follows:

$$ {\displaystyle \begin{array}{c}a=\frac{SUL_{corrected}-{SUL}_{measured}}{HU_{ref}-{HU}_{measured}}\\ {}{SUL}_{corrected}=a\times \left({HU}_{ref}-{HU}_{measured}\right)+{SUL}_{measured}\end{array}} $$

where a (the slope of the linear regression equation observed between liver HU_mean_ and SUL_max_ values) was equal to 0.01037 (Fig. 5b).

$$ {SUL}_{corrected}=0.01037\times \left(55-{HU}_{measured}\right)+{SUL}_{measured} $$

Using liver SUL_max_corrected_ values, there was no longer a correlation between SUL_max_corrected_ and liver HU_mean_ (*R* ^2^ = 2.581e-008, *p* = 0.9976) (Fig. 5c). Furthermore, there were no significant differences between mean SUL_max-corrected_ values of the steatosis versus non-steatotic groups of patients for both interim and EoT PET: 1.98 ± 0.33 versus 2.15 ± 0.27 and 2.04 ± 0.30 versus 2.17 ± 0.31, respectively (Fig. 3b).

### Impact of liver steatosis on the Deauville criteria

Using liver SUL_max corrected_, three DS changes were observed in two patients. Indeed, all steatotic patients with a Deauville score 4 on interim (*n* = 1) and EoT (*n* = 2) PET-examinations moved to DS 3. These examinations corresponded to a 36-year old obese female patient assessed for a Hodgkin lymphoma who received R-BAC (Rituximab-Bendamustine/Aracytine/Cytarabine) as third-line of treatment (patient #108) and a 53-year old overweight male patient assessed for a follicular lymphoma who received R-CHOP as second-line of treatment (patient #146). The first patient is still alive without any sign of lymphoma relapse under maintenance treatment with Ruxolitinib in a phase 2 trial. The second patient died 111 days after his EoT PET-CT examination from a multi-visceral failure secondary to a veno-occlusive disease occurring during an allograft of haematopoietic stem cells. Also, one steatotic patient DS 5 moved to DS 4. Delta SUL_max_ for the first and second patients was −60.5% and −89.9%, respectively. Quantitative data of these patients can be found in Table 4.

**Table 4:** Quantitative data of steatotic patients moving from DS4 to DS3 after SUL_max_ correction

	Patient #	Tumour SUL_max_	Liver SUL_max_	Liver SUL_max_corrected_^a^
iPET	108	2.16	1.92	2.20
EoT PET	108	1.95	1.59	2.11
	146	2.02	1.89	2.58

^a^SUL_max_corrected_ = 0.01037 x (55 – HU_measured_) + SUL_max_measured_

### Effect of steatosis on SUV_max_ values

Taking into account that most of the PET units use SUV_max_ and not SUL_max_ for a routine purpose, SUV_max_ data are also described below.

**Fig. 6:**
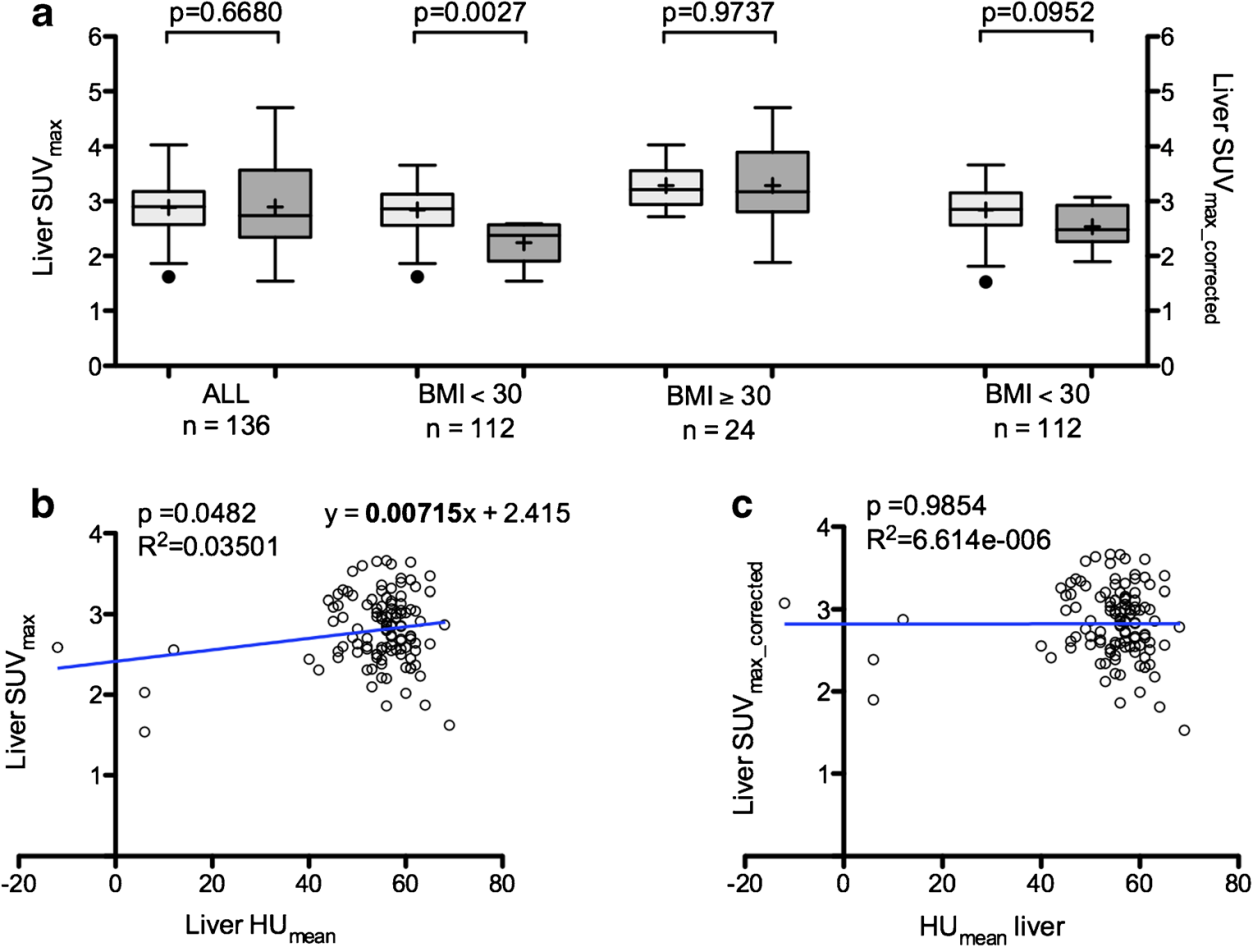
**Impact of steatosis on liver SUV**_**max**_ **in the EoT group.** Liver SUV_max_ values of steatotic and non-steatotic patients before and after correction regarding liver density/steatosis (**a**). Data are shown as Tukey boxplots (lines displaying median, 25th and 75th percentiles; cross represents the mean value). Correlation between liver SUV_max_ values and liver HU_mean_ values of BMI < 30 kg/m^2^ patients before (**b**) and after (**c**) correction regarding liver density/steatosis. EoT: End of Treatment

Considering all patients, mean liver SUV_max_ values were not significantly different between steatotic and non-steatotic groups of patients in both interim and EoT groups: 2.86 ± 0.71 versus 2.82 ± 0.36 (*p* = 0.78) and 2.90 ± 0.84 versus 2.89 ± 0.43 (*p* = 0.67), respectively. However, in the EoT group, when focusing only on normal-weight and overweight patients (BMI < 30 kg/m^2^ patients) (*n* = 112/136 patients, 82.3% of patients), mean liver SUV_max_ values were significantly lower in the steatotic versus non-steatotic group: 2.24 ± 0.40 versus 2.84 ± 0.42. In obese patients (BMI ≥ 30 kg/m^2^), no significant difference was observed (Fig. 6a). This could be explained by higher inconsistences of SUV calculation in obese patients because of the wrong approximation of the ^18^F–FDG volume of distribution in the SUV_max_ equation as compared to SUL_max_. Indeed the mean %differences between SUV_max_ and SUL_max_ were −23.03 and −40.06 in BMI < 30 kg/m^2^ and BMI ≥ 30 kg/m^2^ patients, respectively, with higher ranges in obese patients (additional Fig. 1). The interim group could not be evaluated in the same way because of a limited number of steatotic events in both BMI < 30 kg/m^2^ and BMI ≥ 30 kg/m^2^ groups (only three cases in each).

Liver SUV_max_ values were significantly correlated with liver HU_mean_ values in EoT PET examinations of BMI < 30 kg/m^2^ patients (*R* ^2^ = 0.03501, *p* = 0.0482) (Fig. 6b). The slope of the linear regression equation observed between liver HU_mean_ and SUV_max_ values was equal to 0.00715. Moreover, for all examinations considered, the median value of liver HU_mean_ was 56 HU. This value was then taken as the value of reference: HU_ref_. Thus, using the same method as for SUL_max_, the equation for the adjustment of SUV_max_ in BMI < 30 kg/m^2^ patients would be:

$$ {SUV}_{corrected}=0.00715\times \left(56-{HU}_{measured}\right)+{SUV}_{measured} $$

Using liver SUV_max_corrected_ values, there were no significant differences between mean SUV_max_corrected_ values of the steatotic versus non-steatotic groups of BMI < 30 kg/m^2^ patients for EoT PET: 2.53 ± 0.41 versus 2.84 ± 0.43 (Fig. 6a). Furthermore, there was no longer a correlation between SUV_max_corrected_ and liver HU_mean_ (*R* ^2^ = 6.614ee-006, *p* = 0.9854) (Fig. 6c).

As for liver SUL_max_corrected_, using liver SUV_max_corrected_ patient #146 with DS4 moved to DS3. Patient #108 was an obese patient and, therefore, could not be computed in the same way. Of note, one patient DS4 moved to DS5.

## Discussion

The prevalence of NAFLD increases by a factor of 4.6 in obese people, defined as those with a body-mass index of at least 30 [12]. Considering the increasing prevalence of obesity among adults and children [13], steatosis may soon become a greater issue. In the present study involving 227 lymphoma patients over a period of 1 year, hepatic steatosis was observed in 11.9% of the patients at some point during their baseline or post-treatment evaluation. This is less than the prevalence observed in the general population and could certainly be explained by the cut-off of 42 HU used to discriminate steatotic versus non-steatotic patients. Indeed, the use of this cut-off value enables the diagnosis of macrovesicular steatosis of 30% or greater. Therefore, mild macrovesicular steatosis (<30%) was not considered in the present study. Yet, by virtue of higher fatty than hepatic parenchymal density, this cut-off value permits an easy recognition of steatosis on the non-contrast CT component of a PET/CT and can easily be performed in clinical practice. Interestingly, post-treatment hepatic steatosis was not apparently related to the type of chemotherapy regimen, nor to the time-course of treatment and therefore does not explain the variability of liver ^18^F–FDG uptake previously observed in patients with DLBCL and HL [14].

^18^F-FDG PET/CT has already been explored in the context of hepatic steatosis but never in a population of lymphoma patients. These previous studies led to contradictory results with positive [15], negative [16] or even no relationship [17] between hepatic steatosis and liver SUVs. This could potentially be explained by other factors influencing the liver uptake, such as BMI, which is definitely linked to hepatic steatosis [18], not being considered. However, Lin’s results were in accordance with ours [16] and in the same line Abele et al., observed lower SUV_mean_ in steatotic patients even though statistical significance was not reached [17]. The study showing a positive relationship between steatosis and SUV_max_ did not actually take into account patients BMI, and; therefore, these results were certainly biased [15]. In our study, BMI and BGL appeared to be statistically different between steatotic and non-steatotic groups of patients whereas other parameters described as potentially affecting hepatic uptake {age, sex, treatment, time course of treatment [14, 19–21]} were not. Concerning BGL, a proportional relationship with ^18^F–FDG liver uptake has been shown, even for blood glucose in line with EANM recommendations [22]. However, in our study, liver SUL_max_ values were not significantly linked to BGL recorded at injection time for either interim or EoT PET scans. To take into account the BMI, which is the main confounding parameter regarding steatosis, we used liver SUL_max_ values instead of the recommended SUV_max_ values for the determination of Deauville Scores [11]. However, according to the EANM procedure guidelines for tumour imaging [22], SUL is a recommended quantitative measure of ^18^F-FDG uptake and, in our study, SUL_max_ values gave the same DS as SUV_max_ values in almost all cases except in two examinations among 215 interim and EoT PET examinations (0.9%). Furthermore, these corresponded to changes between DS 4 and 5 or DS 2 and 3, meaning no change between responder versus non-responder status. These results suggest that either SUV_max_ or SUL_max_ can be used to score patients with relatively consistent results. However, the use of SUL_max_ has the advantage of giving the opportunity to reveal and potentially take into account parameters other than BMI that could influence the liver uptake, such as steatosis in the present case.

Liver SUL_max_ values were significantly lower in the presence of hepatic steatosis (defined as a mean liver density ≤ 42 HU) in both interim and EoT PET scans with an average decrease of 29.5% and 29.9%, respectively (Fig. 3a). Liver SUV_max_ values were also significantly lower in the presence of hepatic steatosis in EoT PET scans of normal-weight and overweight patients (BMI < 30 kg/m^2^) with an average decrease of 21.1% (Fig. 6a). The same results were not observed in obese patients (BMI ≥ 30 kg/m^2^) underlining the fact that this group clearly benefit from the use of SUL instead of SUV values that do not take into account the absence of ^18^F–FDG uptake in adipose tissue. One could thus expect a possible overestimation of the DS in steatotic patients from DS3 to DS4 with the risk of inducing erroneous modifications in patients’ management. Indeed, being classified as a non-responder (DS4) on an EoT PET usually prompts the use of a subsequent line of chemotherapy. Another important finding is that liver HU_mean_ values were the only independent factor correlated to liver SUL_max_ values in interim and EoT groups (Table 3) which allowed us to successfully correct liver SUL_max_ values by using a graphical method (Fig. 5) based on the slope of the calculated linear regression. Importantly, the application of this liver SUL_max_ correction led to the reclassification of all steatotic patients scored DS4 to DS3. Given the small number of steatotic patients scored DS4 on interim and EoT PET scans in our series, it is not possible to assess the clinical impact and veracity of these changes with respect to therapeutic intervention or patient outcome. Larger and more homogeneous studies are needed to substantiate the advantage of correcting the liver uptake of steatotic patients initially scored DS4. Our findings could also be confirmed in trials with homogeneously treated patients for which central reviewing has been performed. For instance, in the IELSG-26 study in patients with primary mediastinal DLBLC [23], the rate of DS4 was 24/115 (21%) and in a recent trial in advanced HL [24], it was 144/1119 (12.9%). However, at the present time, attention should be paid to the interpretation of the PET examinations scored DS4 with a percentage difference between the target lesion and the liver background lower than 30%, which is the average decrease in our series, and steatosis should be sought for on the CT part of the PET/CT examination to avoid erroneous alteration of patient’ management. When steatosis is depicted, the application of a correction as described in the results section or the use of delta SUL should be considered.

## Conclusion

In a population of 227 lymphoma patients referred for interim and EoT PET scans over a 1-year period, prevalence of steatosis was 11.9%. Irrespective of patient’s BMI, blood glucose level and the type of chemotherapy regimen, liver SUL_max_ values were significantly lower in steatotic patients as compared to non-steatotic patients, with an average decrease of almost 30%. This is actually a theoretical but not a practical issue in most patients but should be recognised and corrected in appropriate cases, namely, for those patients scored DS4 with a percentage difference between the target lesion and the liver background lower than 30%.

## Electronic supplementary material

Supplemental Fig. 1 Percentage difference between liver SUV_max_ and liver SUL_max_ values. (GIF 11 kb)

High resolution image (TIFF 3342 kb)
