# Supplementary figures and images for: Assessment of alteration in liver 18F–FDG uptake due to steatosis in lymphoma patients and its impact on the Deauville score
Source: Eur J Nucl Med Mol Imaging. 2017 Dec 26;45(6):941–50. doi: 10.1007/s00259-017-3914-y (PMC5915498; doi:10.1007/s00259-017-3914-y)

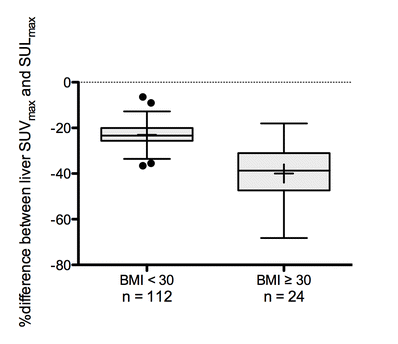

Supplement: Supplementary file 1 — Percentage difference between liver SUVmax and liver SULmax values. (GIF 11 kb) [file 259_2017_3914_Fig7_ESM.gif]

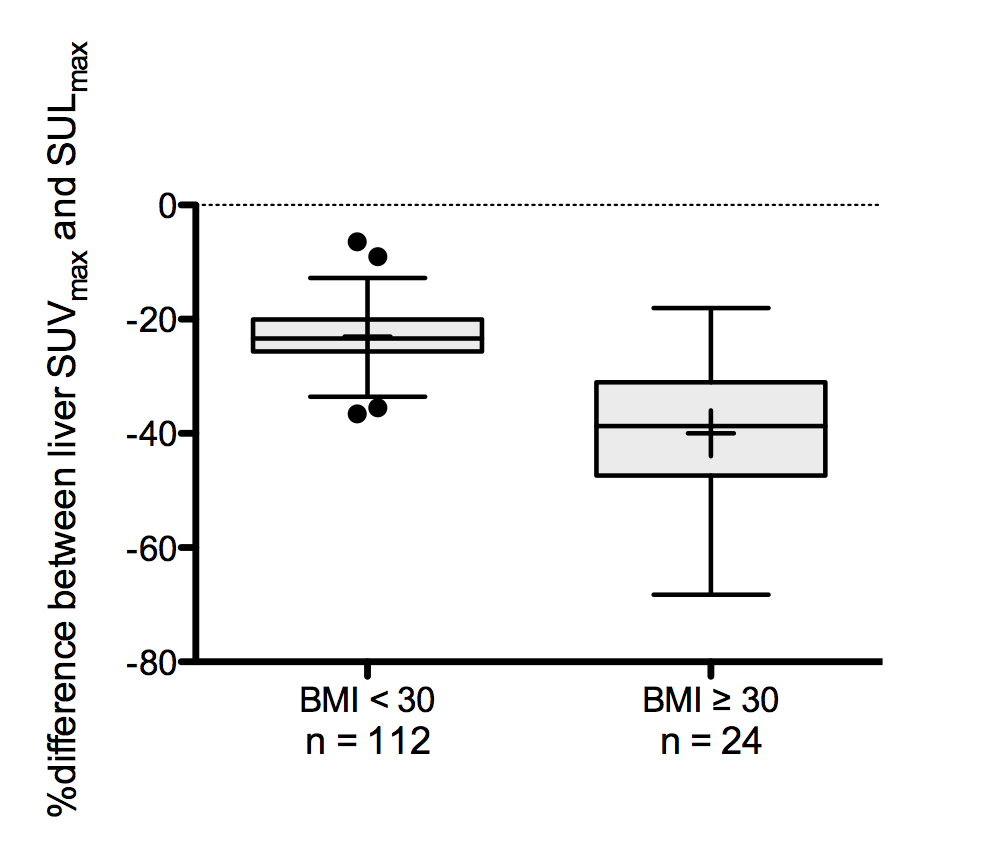

Supplement: Supplementary file 2 — High resolution image (TIFF 3342 kb) [file 259_2017_3914_MOESM1_ESM.tiff]
